# Enlightening the bimetallic effect of Au@Pd nanoparticles on Ni oxide nanostructures with enhanced catalytic activity

**DOI:** 10.1038/s41598-023-29679-6

**Published:** 2023-02-24

**Authors:** Luca Bruno, Mario Scuderi, Francesco Priolo, Luigi Falciola, Salvo Mirabella

**Affiliations:** 1 Dipartimento di Fisica e Astronomia “Ettore Majorana”, Università degli Studi di Catania, via S. Sofia 64, 95123 Catania, Italy; 2 CNR-IMM (Catania Università), via S. Sofia 64, 95123 Catania, Italy; 3 CNR-IMM, VIII Strada 5, 95121 Catania, Italy; 4 Dipartimento di Chimica, Università degli Studi di Milano, Via Golgi 19, 20133 Milan, Italy

**Keywords:** Nanoparticles, Synthesis and processing, Electrochemistry

## Abstract

Bimetallic decoration of semiconductor electrodes typically improves catalytic and sensing performances because of a well-claimed synergistic effect. A microscopic and quantitative investigation of such an effect on energy bands of semiconductor can be really useful for further exploitation. Au, Pd and Au@Pd (core@shell) nanoparticles (10–20 nm in size) were synthesized through chemical reduction method and characterized with scanning and transmission microscopy, Rutherford backscattering spectrometry, cyclic voltammetry electrochemical impedance spectroscopy and Mott–Schottky analysis. The nanoparticles have been used to decorate Ni-based nanostructured electrodes with the aim to quantitatively investigate the effect of decoration with mono or bimetallic nanoparticles. Decorated electrodes show higher redox currents than bare ones and a shift in redox peaks (up to 0.3 V), which can be ascribed to a more efficient electron transport and improved catalytic properties. These effects were satisfactorily modeled (COMSOL) employing a nano Schottky junction at the nanoparticle–semiconductor interface, pointing out large energy band bending (up to 0.4 eV), space charge region and local electric field (up to ${10}^{8}\mathrm{ V }{\mathrm{m}}^{-1}$) in bimetallic decoration. Sensing test of glucose and H_2_O_2_ by decorated Ni oxide electrodes were performed to consolidate our model. The presence of bimetallic nanoparticles enhances enormously the electrochemical performances of the material in terms of sensitivity, catalytic activity, and electrical transport. The modification of energy band diagram in semiconductor is analyzed and discussed also in terms of electron transfer during redox reactions.

## Introduction

Bimetallic nanoparticles (NPs) have attracted enormous interest in the past decade due to their intriguing physical and chemical properties, and their applications in many fields of materials science (catalysis, photocatalysis, optics, sensing, and nanomedicines)^1–5^. Indeed, the addition/substitution of one or more chemical elements to a metallic surface increases the possible bonding geometries of surface adsorbates and simultaneously changes the electronic structure at surface^6^. Generally, bimetallic NPs can be classified according to their mixing pattern (chemical ordering) and geometric structure. Two main categories of NPs can be identified: core–shell (or core@shell) and nanocomposite bimetallic NPs, with an ordered atomic arrangement in the first case and random mixed atoms in the second one^2^. Independently of the ordering, a certain improvement of catalytic and sensing performance is observed and typically explained inferring a *synergistic* effect^7^. In fact, most fundamental properties of NPs cannot be described as extrapolation of bulk properties. From the theoretical perspective, bimetallic NPs provide ideal test bench for the development of novel theoretical concepts and techniques and present a series of questions of fundamental interest^8^.

Several theoretical investigations studied this synergistic effect on surface adsorption and chemical reactions^9–14^. When a foreign metal atom is added to a metal host, a key change occurs in *d*-band position and filling, caused by local bonding alteration to accommodate the foreign atom. The number of *d*-electrons is typically affected by these changes, and its variation leads to a change in reactivity of bimetallic nanoparticles^15^. From the experimental point of view, the effect of bimetallic NPs induces a sensible boost in terms of sensitivity^16,17^, catalytic activity^11,14,18^, and electrical properties^19,20^. However, most experimental papers, beyond measuring the bimetallic effect and invoking an unspecified synergistic effect, miss any attempt to microscopic characterize and model it^21^. A deep understanding of the relationship between catalytic outcome and synergistic effect in bimetallic NPs could help in developing more efficient sensors with low cost, enhanced activity, and high selectivity^21^.

Among the different applications of bimetallic NP, electrochemical sensors are largely involved to exploit the synergistic effect thanks to their simplicity, low-cost fabrication, and ease to be minimized, leading to higher sensitivity and selectivity^22–30^. In particular, core–shell bimetallic Au@Pd NPs are observed to have superior synergistic effects^11–14,26,31^. In most cases, the effect of decoration with bimetallic NPs is evaluated on flat or bulk substrates^12,26,32–34^. The enhanced electrocatalytic activity and electrochemical stability of bimetallic NPs were exploited for electrochemical enzyme-free sensing achieving high sensitivity and selectivity^12,34,35^. The presence of NPs on nanostructured substrates can link the advantages of nanostructures (large surface area and/or quantum size effect) with the specificity of synergistic effects.

In this work, we experimentally investigate the decoration of a Ni oxide nanostructure with NPs of Au, Pd and Au@Pd (Au core, Pd shell). Bimetallic NPs give a key improvement in catalytic and sensing performances of Ni-based electrodes. Morphological, chemical, and electrochemical characterizations were used with a multiphysics simulation in order to model the synergistic effect with energy band bending at the metal/semiconductor interface.

## Methods

### Colloidal Au, Pd and Au@Pd dispersion

**Figure 1:**
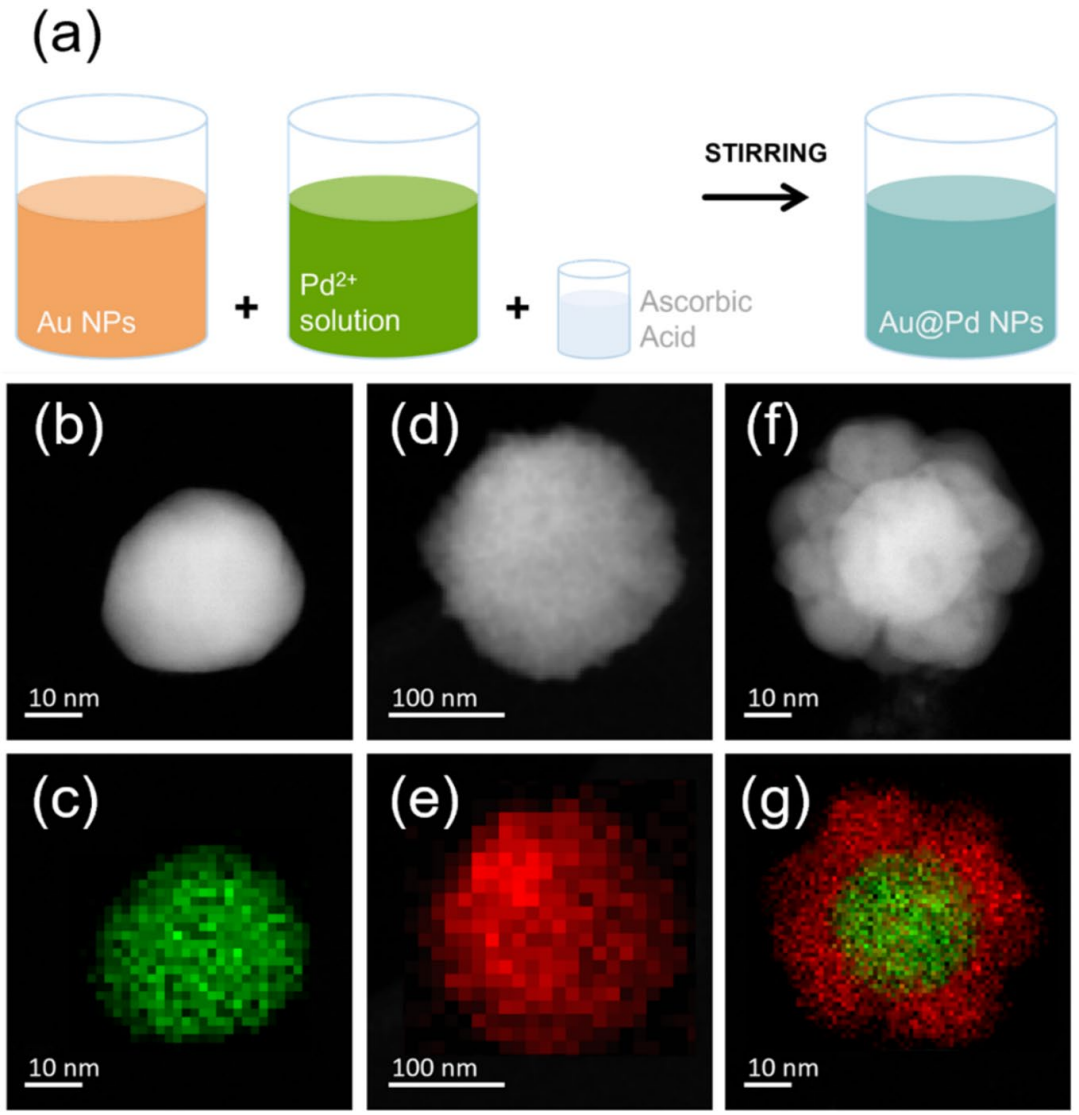
(**a**) Schematic representation of Au@Pd core–shell NPs; STEM (**b**,**d**,**f**) images and EDX (**c**,**e**,**g**) elemental maps of representative Au (**b**,**c**), Pd (**d**,**e**) and Au@Pd (**f**,**g**) NPs.

Dispersions containing Au or Pd or Au@Pd NPs were produced through a green chemical reduction method at room temperature using ascorbic acid (AA) as reducing agent^36^, instead of the most common trisodium citrate^37–40^. Monometallic Au and Pd NPs were synthesized by adding 30 $\mathrm{\mu L}$ of 33 mM AA in 30 mL of 0.12 mM ${\mathrm{HAuCl}}_{4}$ (Sigma-Aldrich, St. Louis, MO, USA, $\ge$ 99.9%) and 0.9 mM ${\mathrm{PdCl}}_{2}$ (Sigma-Aldrich, St. Louis, MO, USA, $\ge$ 99.9%) aqueous solutions, respectively. The solutions were stirred for 5 min. Concerning Au@Pd NP synthesis, 10 mL of Au NPs colloidal solution and 30 mL of ${\mathrm{PdCl}}_{2}$ aqueous solution were mixed with 50 $\mathrm{\mu L}$ of AA (Sigma-Aldrich, St. Louis, MO, USA) and stirred for 5 min (Fig. 1a). The dispersions were used without any further cleaning.

### Decorated Ni oxide electrode (NOE)

Fluorine-doped Tin Oxide (FTO) substrates ($1 \times 2\mathrm{ c}{\mathrm{m}}^{2}$, resistivity $\sim 13\Omega /\mathrm{sq}$, Sigma-Aldrich, St. Louis, MO, USA) were rinsed with acetone, isopropyl alcohol, and deionized water (MilliQ, 18 $\mathrm{M\Omega cm}$) and dried under ${\mathrm{N}}_{2}$ gas flow. Onto cleaned substrates $\mathrm{Ni}{\left(\mathrm{OH}\right)}_{2}$ nanostructures were obtained by chemical bath deposition (CBD), through a bain-marie configuration at 50 °C^41^. Solution for CBD was prepared by mixing 0.42 M ${\mathrm{NiSO}}_{4}\cdot 6{\mathrm{H}}_{2}\mathrm{O}$ (Merck, Darmstadt, Germany, 98%), 0.07 M ${\mathrm{K}}_{2}{\mathrm{S}}_{2}{\mathrm{O}}_{8}$ (Alfa Aesar, Kandel, Germany 97%) and 3.5 wt% ammonia (Merck, Darmstadt, Germany, 30–33 wt% ${\mathrm{NH}}_{3}$ in ${\mathrm{H}}_{2}\mathrm{O}$). Once extracted, the samples were rinsed with deionized water (MilliQ, 18 $\mathrm{M\Omega cm}$) and dried in ${\mathrm{N}}_{2}$ gas and annealed at 350 °C for 60 min in Ar followed by 60 min in forming gas (FG, $\mathrm{Ar}:{\mathrm{H}}_{2}$ 95:5 mixture). This process leads to a metallic Ni nanostructure because of reducing annealing ambient^42^. The metallic Ni nanostructure is then decorated via spin coating (500 rpm, 60 s) by using 50 $\upmu$L of the mono or bimetallic NPs dispersion as prepared. The samples are dried on a hot plate at 80 °C for 10 min. Finally, the Ni oxide electrode (NOE) were obtained by several cycles of cyclic voltammetry (CV, in 0.1 M NaOH, scan rate of $0.05\mathrm{ V }{\mathrm{s}}^{-1}$) between − 0.3 and 0.9 V vs SCE, leading to surface oxidation of Ni^43^. Decorated samples are labelled according to NP type onto NOE (e.g., Au-NOE refers to NOE decorated with Au NPs).

### Analytical techniques

UV–Vis spectroscopy was performed on Au solution using a Varian Cary 500 (Agilent technologies, California, USA) double beam scanning UV/Vis/NIR spectrophotometer (scan range 350–800 nm).

The elemental composition of NPs in the dispersions was evaluated on a Si wafer coated with NPs (via spin coating) by Rutherford backscattering spectrometry (RBS, 2.0 MeV ${\mathrm{He}}^{+}$ beam at normal incidence) with a 165° backscattering angle by using a 3.5 MV HVEE Singletron accelerator system (High Voltage Engineering Europa, Netherlands). RBS spectra were analyzed by using XRump software^44^.

Surface morphology was analyzed by using a Scanning Electron Microscope (SEM, Gemini field emission SEM Carl Zeiss SUPRA 25, FEG-SEM, Carl Zeiss Microscopy GmbH, Jena, Germany).

Transmission electron microscopy (TEM) analyses of Au, Pd and Au@Pd NPs dispersed on a TEM grid were performed with a Cs-probe-corrected JEOL JEM ARM200F microscope at a primary beam energy of 200 keV operated in scanning TEM (STEM) mode and equipped with a 100 mm^2^ silicon drift detector for energy dispersive X-ray (EDX) spectroscopy. For EDX elemental mapping, the Au M and Pd L X-rays signals were collected by scanning the same region multiple times with a dwell time of 1 ms. TEM images and EDX spectra were analyzed by using DigitalMicrograph^®^ software^45^.

Electrochemical measurements were carried out at room temperature by using a VersaSTAT 4 potentiostat (Princeton Applied Research, USA) and a three-electrode setup with a platinum counter electrode, a saturated calomel electrode (SCE) as reference electrode, and our samples as working electrode, without purging any inert gas. 0.1 M NaOH (Sigma Aldrich, St. Louis, MO, USA) was used as supporting electrolyte. Cyclic voltammetry (CV) curves were recorded at a scan rate of 50 $\mathrm{mV }{\mathrm{s}}^{-1}$ scanning the potential from − 0.3 to 1.0 V vs reference electrode (SCE). Electrochemical impedance spectroscopy (EIS) was performed at the oxidation peak potential with a superimposed 5 mV sinusoidal voltage in the frequency range ${10}^{4}\div{10}^{-1}$ Hz. Mott–Schottky (M–S) analyses were conducted on bare and decorated NOE samples in the potential range $-0.5\div1$ V vs SCE, at 1000 Hz frequency. Chronoamperometry (CA) analysis was employed to study the response of the samples to successive additions of different amounts of glucose (d-(+)-glucose, Sigma-Aldrich, St. Louis, MO, USA) and ${\mathrm{H}}_{2}{\mathrm{O}}_{2}$ (30 w/w% ${\mathrm{H}}_{2}{\mathrm{O}}_{2}$ in ${\mathrm{H}}_{2}\mathrm{O}$, Sigma-Aldrich, St. Louis, MO, USA) to the 0.1 M NaOH solution.

A simulation of semiconductor energy bands, electron concentration, and electric field at the metal/semiconductor interface has been carried out by *COMSOL Multiphysics software*^46^.

## Results and discussion

### Morphological and elemental characterization

The synthesis of bimetallic Au@Pd NPs is shown in Fig. 1a. The presence of Au, Pd and Au@Pd NPs within the colloidal dispersion was checked with UV–Vis spectroscopy (Figure S1a). Au spectrum shows a narrow and sharp plasmonic peak (centered at around 530 nm) which is a clear indication of the presence of stable NPs in colloidal form^39^, while no peak can be observed in the case of Pd colloidal solution (it is known that colloidal dispersions of Pd NPs synthesized with AA do not exhibit a well-defined absorption band^36^). On the other hand, in the Au@Pd dispersion, the Au peak at 530 nm is drastically reduced because of the contemporary presence of both Au and Pd, suggesting that Au atoms are confined in the core of the NPs whereas Pd atoms are on the surface^36,47^. RBS analyses (Fig. S1b) confirm the presence of Au (peak at 1.8 MeV) and Pd (peak at 1.7 MeV) onto Si as expected. RBS was used to confirm the effective presence of mono and bimetallic NPs after the decoration of a flat Si substrate, since Au and Pd amounts are proportional to the peak area^48^. In the case of Au@Pd NPs, the amount of Au and Pd is $7.0\times {10}^{15}\mathrm{ at c}{\mathrm{m}}^{-2}$ and $1.6\times {10}^{15}\mathrm{ at c}{\mathrm{m}}^{-2}$, respectively. Moreover, these peaks are wider in energy than in monometallic cases. Such a feature indicates the presence of 3D agglomerates of NPs spread onto the surface with a NP mean size higher than those of mono-metallic case.

STEM pictures and EDX color maps of representative Au, Pd and Au@Pd NPs are shown in Fig. 1b–g. Au NPs have a rounded-like shape with a quite uniform diameter of about $(20\pm 3)$ nm and some bumps due to their crystalline structure (Figure S2a). Even if Pd NPs also show a spherical shape (with size ranging from few to hundreds of nanometers, Fig. S2b) the clear and ubiquitous presence of small Pd grains tells us that Pd NPs are always composed of these very small grains. STEM image of Au@Pd NPs reveals the presence of a Au core ($\sim$ 25 nm) and Pd shell ($\sim$ 10 nm) made of smaller Pd clusters (Fig. 1f), with a core–shell like structure. This result is confirmed with STEM EDX spectroscopy data. Indeed, elemental maps in Fig. 1g and the corresponding line-scan of Fig. S2c show clear evidence of the core–shell configuration.

**Figure 2:**
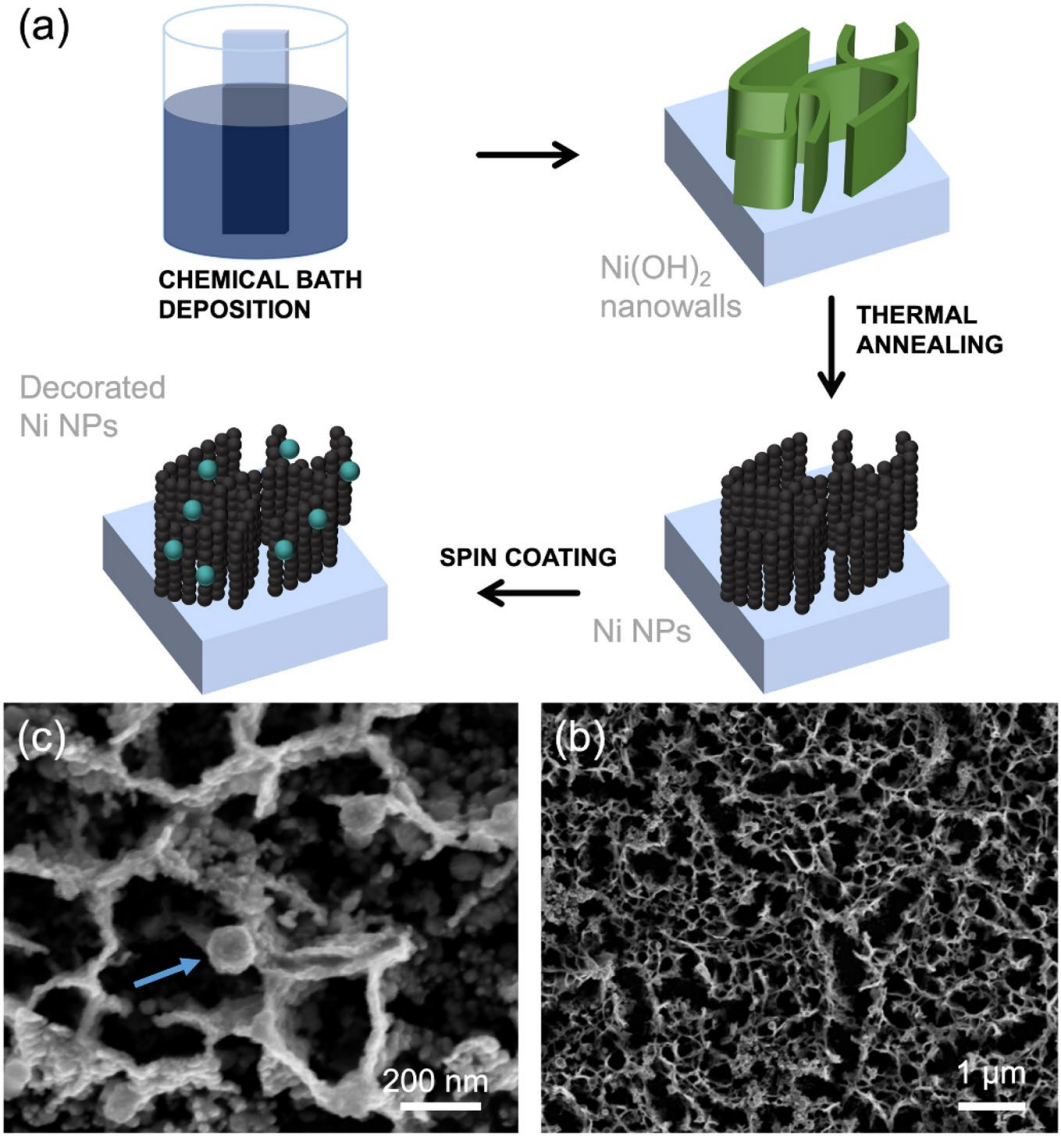
(**a**) Schematic representation of synthesis and decoration of Ni NPs; (**b**) SEM image of porous Ni NPs; (**c**) high magnification SEM image of Au@Pd NPs anchored on the top of Ni NPs (blue arrow).

Ni-based electrode was obtained by a three-step synthesis shown in Fig. 2 (see Fig. 2a). The 50 °C CBD leads to a $\mathrm{Ni}{\left(\mathrm{OH}\right)}_{2}$ nanowalls structure with thin ($\sim$ 20 nm) interconnected sheets perpendicular to substrate. The reducing thermal process at 350 °C (for 1 h in Ar and 1 h in FG) leads to a structural and chemical transformation in which nanowalls are converted into chain-like clusters of metallic Ni NPs (20 nm large) (Fig. 2b)^43^. Such Ni NPs were decorated (Fig. 2c) with mono- and bimetallic NPs by spin coating with colloidal dispersion and drying on hot plate at 80 °C for 20 min.

### Electrochemical modification and characterization

**Figure 3:**
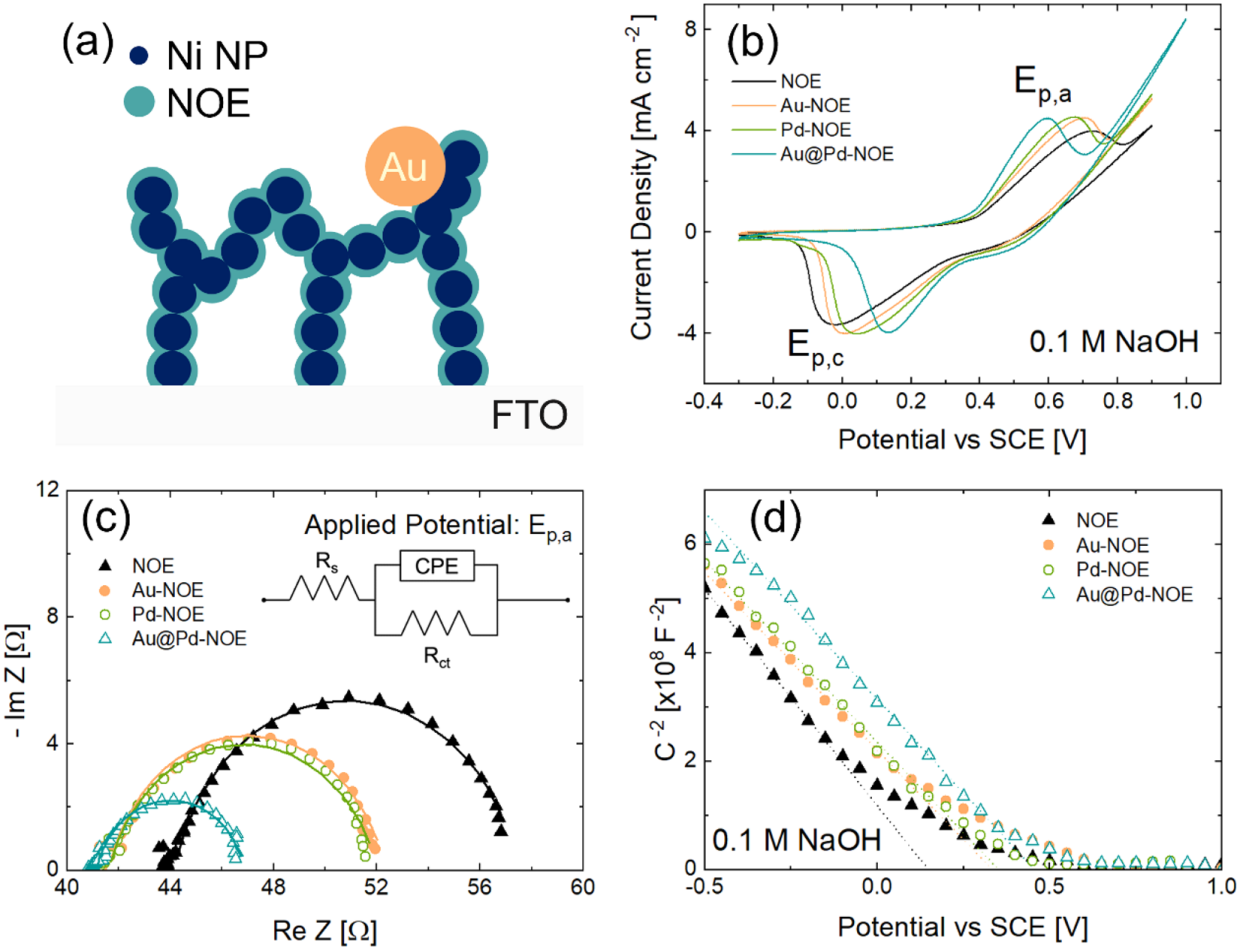
(**a**) Scheme of a cross section of decorated NOE. Electrochemical characterization of bare and decorated electrodes: (**b**) cyclic voltammograms, (**c**) Nyquist plots from EIS analyses, (**d**) Mott–Schottky plots. The bimetallic NP decoration induces a larger variation in comparison to the monometallic NP case.

The bare or decorated metallic Ni NPs were then modified by electrochemical methods in order to form a thin layer of Ni oxide/hydroxide through several cycles of cyclic voltammetry (CV, in 0.1 M NaOH, scan rate of $0.05\mathrm{ V }{\mathrm{s}}^{-1}$) between − 0.3 and 0.9 V vs SCE (Fig. 3a)^43,49^. The surface oxidation of Ni is the last step to produce Ni oxide electrodes (NOE) consisting of a metallic Ni skeleton covered by a thin semiconductor Ni oxide/hydroxide, possibly decorated with mono- or bimetallic NPs.

Figure 3b displays the cyclic voltammograms of bare electrode compared to that decorated with Au, Pd, and Au@Pd NPs. CV curves were recorded in 0.1 M NaOH at a scan rate of $0.05\mathrm{ V }{\mathrm{s}}^{-1}$ between − 0.3 and 0.9 V vs SCE, for several cycles, until stable voltammograms are obtained. This leads to the oxidation of surface Ni into a $\sim 3\mathrm{ to }4$ nm $\mathrm{NiOOH}/\mathrm{Ni}{\left(\mathrm{OH}\right)}_{2}$ shell uniformly recovering Ni NPs^43,49^. Actually, the CV curves always show a pair of redox peaks around $\mathrm{V}=0.6\div 0.75$ V vs SCE (oxidation peak) and $\mathrm{V}=-0.05\div 0.15$ V vs SCE (reduction peak), caused by the reversible transition ${\mathrm{Ni}}^{2+}/{\mathrm{Ni}}^{+3}$ in alkaline medium^41,50,51^:

1 $$\mathrm{Ni}{\left(\mathrm{OH}\right)}_{2} +{\mathrm{OH}}^{-} \leftrightarrow \mathrm{ NiOOH}+{\mathrm{H}}_{2}\mathrm{O}+{\mathrm{e}}^{-}$$

From a microscopic point of view, the liquid/solid interface tested by electrochemical analysis is composed of used electrolyte (0.1 M NaOH) and $\mathrm{NiOOH}/\mathrm{Ni}{\left(\mathrm{OH}\right)}_{2}$ semiconductor decorated with mono- or bimetallic NPs. The effect of decoration on the redox behavior of Ni electrode will be obtained by comparing the bare semiconductor with decorated one. Compared to bare electrode, the decorated ones present higher peak currents both in oxidation and reduction. In addition, the gap between anodic (${E}_{p,a}$) and cathodic (${E}_{p,c}$) peak potentials ($\Delta {E}_{p}={E}_{p,a}-{E}_{p,c}$) decreases in decorated electrodes, especially in bimetallic case. At the same time, the full width at half maximum (FWHM, calculated by subtracting the background signal to the peak one) of both anode and cathode peaks is also reduced following decoration, especially with bimetallic NPs (Figure S3 shows the redox peaks after background subtraction)^52–54^. To ensure that the presence of NPs affects predominantly the NOE and not the FTO substrate, CVs on Au, Pd, and Au@Pd NP decorated FTO were performed. As shown in Figure S4, neither mono- nor bi-metallic NPs influences the electrochemical performances of ITO since CV curves do not present any difference between each other. This is a clear indication of how NPs decoration is effective in the modification of redox behavior of NOE. It is important to note that, although CV cycling creates a Ni oxide/hydroxide shell at the metal Ni NPs surface, it does not modify the morphology of the nanostructures (a SEM image of NOE after electrochemical measurements is shown in Figure S5).

EIS was employed to study the interfacial properties of electrodes. Details are reported in SI. Figure 3c exhibits the real and imaginary part of impedance (Nyquist plot) of bare and modified electrodes, together with fitting lines assuming a simple Randles circuit (inset)^55^. The decoration induces a dramatic change in semicircular shape of Nyquist plot, reducing the circle diameter (representing the Charge Transfer Resistance (${R}_{ct}$) from $12.5\Omega$(bare NF) to $10-11\Omega$ (Au or Pd–Ni NF), to $5.5\Omega$ (Au@Pd-NF). Fit parameters are reported in Table S1. The decreased peak potentials give an indication of an enhanced capability of bimetallic system to catalyze the electrocatalytic reactions^23,54^. These results prove that decoration accelerates the electron transfer kinetics, especially in the bimetallic case^22,23,56–60^, and well explain the reduction of $\Delta {E}_{p}$ in the CV curves.

To further investigate the mono- and bimetallic decoration on our nanostructured electrode, we performed Mott–Schottky (M–S) analysis (details in SI). The M–S plot typically reports the inverse of squared capacitance (${C}^{-2}$) measured as a function of potential ($E$) applied to the sample, as reported in Fig. 3d. By increasing $\mathrm{E}$, ${C}^{-2}$ goes to zero, indicating a larger and larger capacitance at the solid–electrolyte interface. Such behavior is typical of a *p*-type semiconductor, as the $\mathrm{Ni}{\left(\mathrm{OH}\right)}_{2}$ is^61,62^. The so-called flat band potential, the intercept with *x*-axis (${E}_{FB}$)^63–69^, tells the potential where saturation of $C$ occurs (Table 1). In a planar semiconductor electrode, after proper correction with open circuit potential (${E}_{OC}$), ${\Delta {E}_{M-S}=E}_{FB}-{E}_{OC}$ represents the energy band bending at equilibrium (flat Fermi energy, electrode to electrolyte)^70^. The energy band bending results from the alignment of the Fermi level of bare or modified semiconductor surface and the redox potential of electrolyte^71^. With decoration, there is a clear shift of ${\mathrm{E}}_{\mathrm{FB}}$ towards more positive potential, up to 0.4 V in bimetallic case (Table 1). Even if our electrodes are not planar but nanostructured semiconductor, such evidence points out a key difference in energy band bending due to mono- and bimetallic decoration. A larger value of ${E}_{FB}$ points out that a more positive potential is needed to saturate the capacitance. This datum helps the modeling of bimetallic effects, as follows.

**Table 1:** Values of flat band potential, open circuit potential and energy barrier for bare and decorated samples.

Sample	${E}_{FB}$[V vs SCE]	${E}_{OC}$[V vs SCE]	$\Delta {E}_{M-S}$[V]
NOE	0.150	0.065	0.085
Au-NOE	0.335	0.052	0.283
Pd-NOE	0.352	0.059	0.293
Au@Pd-NOE	0.455	0.047	0.408

### Modeling the bimetallic decoration effect

**Figure 4:**
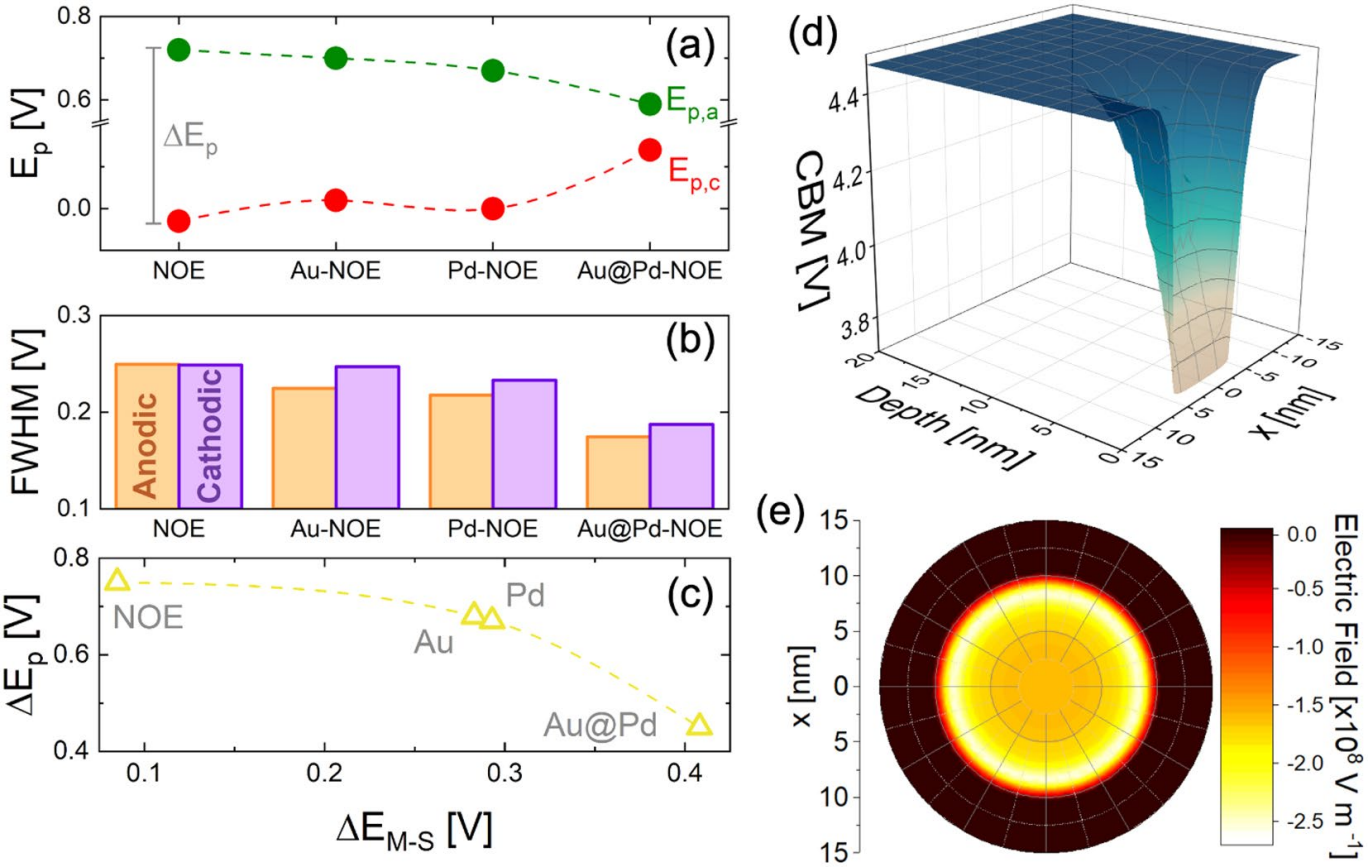
Variation of (**a**) ${E}_{p,a}$ and ${E}_{p,c}$ as a function of metal NP decoration (the peak separation between the two peaks positions in labelled as $\Delta {E}_{p}$), (**b**) FWHM of the anodic and cathodic peaks; (**c**) relation between $\Delta {E}_{p}$ and $\Delta {E}_{M-S}$; COMSOL simulations of (**e**) conduction band minimum (CBM) of Au@Pd-NOE sample, and (**f**) electric field map under a circular Au@Pd dot.

Qualitative and quantitative information can be extracted from electrochemical analytical data. The potential of oxidation (${E}_{p,a}$) and reduction (${E}_{p,c}$) peaks are reported in Fig. 4a for each sample. The position of a voltammetric peak carries information on both thermodynamics and kinetics of the electrochemical process taking place (Eq. 1 in our case)^71–73^. The activation energy for electrode oxidation (or reduction) is provided by applying the electrical potential to the electrode. It should be noted that in our NOE the metallic skeleton provides an effective bias of the catalytic surface, reducing any potential drop, in a fashion similar to what previously modelled^43^. As the activation barrier increases, the electrochemical irreversibility of the system increases and an overpotential is required for the reaction to take place, shifting the oxidation (reduction) peak to higher (lower) electrical potential. Figure 4a represents the catalytic action of NP decoration, by reducing the activation barrier for Eq. (1). It is noteworthy the bimetallic effect which overcomes that of monometallic decoration, strongly reducing the ${\Delta E}_{p}$*.*

Figure 4b reports the FWHM for all the peaks, evidencing a clear shrinking trend with decoration. A catalytic reduction of activation energy results also in a reduction of FWHM, and of both anodic and cathodic peaks, making more and more favorable the electron transfer at solid–electrolyte interface.

Figure 4c clarifies the relation among the $\Delta {E}_{p}$ and $\Delta {E}_{M-S}$, as obtained from Fig. 3. As the energy band bending grows (in bimetallic decorated sample), the energy separation among oxidation and reduction peaks decreases, index of a strong catalytic effect compared to bare sample. The mono- or bimetallic decoration increases the energy band bending, because of electron spillover effects, leading to space charge regions and a potentially very high, localized electric field^19,40^.

To compute and visualize the effect (on electric field and energy band position) of putting a metal NP on a $\mathrm{Ni}{\left(\mathrm{OH}\right)}_{2}$ nanostructure, COMSOL^46^ simulations were performed assuming a single metal circular dot (20 nm large) placed onto the semiconductor (Figure S6a) and simulating a nano Schottky junction^18,40,70^. The simulation does not consider surface defects or temperature dependence and takes into account the experimentally extracted Mott–Schottky band bending (Table 1). Actually, the M–S measurement returns an average behavior of samples and not a local quantification of energy band bending at the decoration site. Despite these limits, the simulation helps to understand the catalytic effect of mono- and bimetallic decoration. The *p*-type semiconductor is greatly enriched in electrons (spillover effect) below the metallic dot (Fig. S6), which means a downward bending of its energy bands. The energy map of the conduction band minimum (CBM) for Au@Pd decorated sample as a function of depth and distance from NP center is reported in Fig. 4d. The downward bending of CBM extends almost 5 nm within semiconductor, denoting a giant electric field under the NP, pointing towards the bulk. Considering our case in which the Ni oxide is confined in a thin shell (3–4 m) on a metal core in our NOE, it is reasonable to hypothesize that the whole nanostructure is depleted of electrons (pushed towards the surface by a giant built-in electric field). The 2D map of electric field at decoration site is shown in Fig. 4e for the bimetallic case, revealing intensity up to ${10}^{8}\mathrm{ V }{\mathrm{m}}^{-1}$. A peculiar “halo effect” comes because of the largest potential gradient at the NP edge^40^. A comparison of band bending and electron concentration at decoration site between Au and Au@Pd decoration is presented in Fig. S6b,c. The bimetallic decoration induces a 10 times higher electron concentration in comparison to Au NP decoration, pointing out how the synergistic effect among Au and Pd in core–shell nanostructures can boost a catalytic action of modified electrodes.

**Figure 5:**
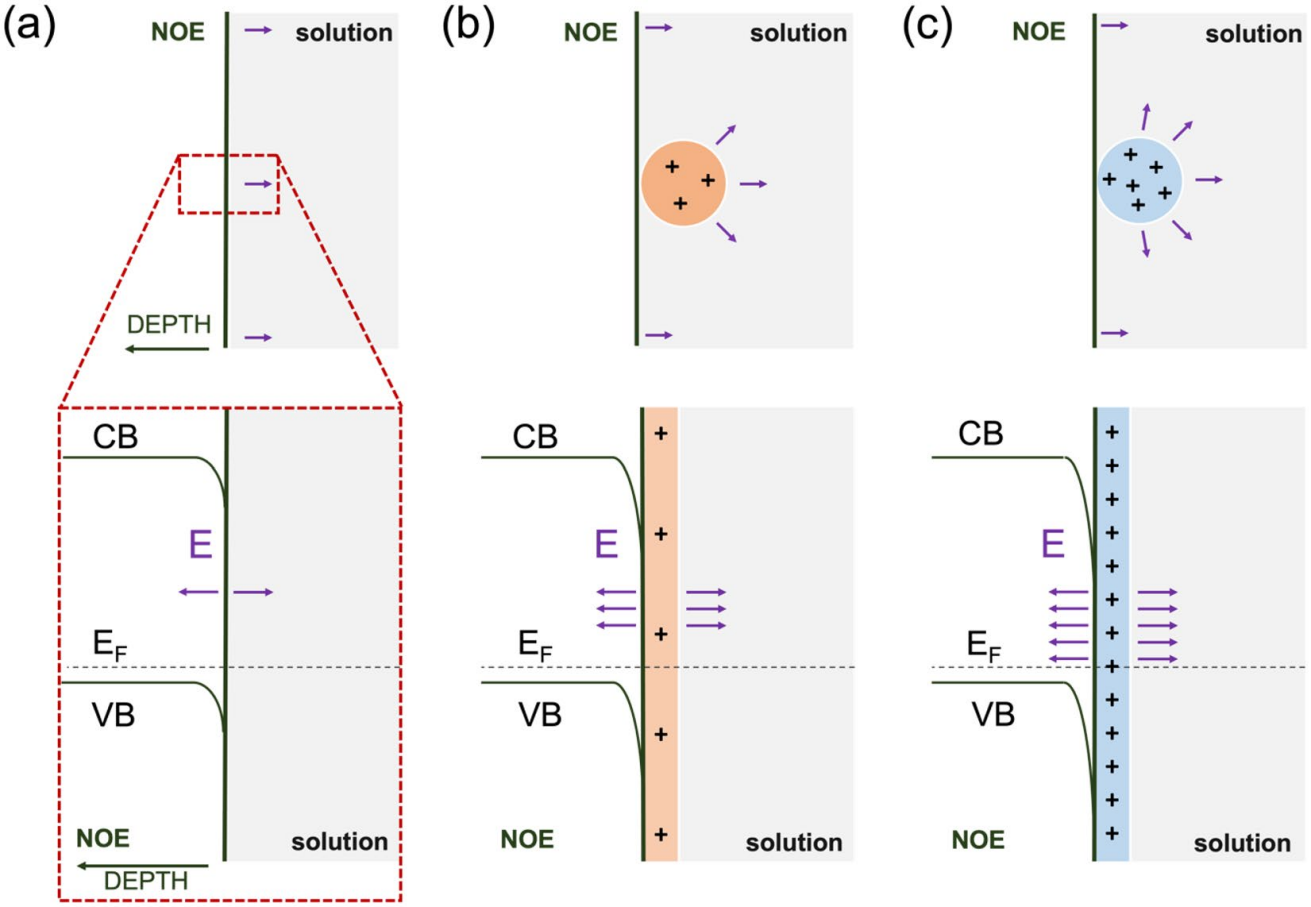
Scheme of energy band bending and local electric field *E* for (**a**) bare, (**b**) Au (or Pd), and (**c**) Au@Pd decorated electrode at solid–liquid interface.

Figure 5 contains a schematic representation of our model of bimetallic effect by exploiting the energy band bending at the decoration site as a consequence of the formation of a nano Schottky junction. Compared to bare electrode (Fig. 5a), the presence of mono- and bimetallic NPs (Fig. 5b,c) induces a larger $\Delta {E}_{M-S}$ which points out a larger energy band bending of semiconductor. The gradient of energy band represents the intensity of localized electric field, which comes from a space charge region. In turns, mono- and bimetallic NP decoration leads to accumulation of immobile positive charges at the solid–electrolyte interface, with greatest extent in case of bimetallic case. In this scenario, the large electric field at decoration site should lead to a net local charge imbalance, with electron accumulation in the Ni oxide at electrode side, and ${\mathrm{OH}}^{-}$ ions buildup at electrolyte side. The increased concentration of ${\mathrm{OH}}^{-}$ ions close to the electrode surface makes more favorable the conversion of $\mathrm{Ni}{\left(\mathrm{OH}\right)}_{2}$ to $\mathrm{NiOOH}$ (Eq. 1) during the anodic scan of the potential. The accumulation of electrons, on the other side, facilitates the electrode reduction during the cathodic scan. In bimetallic NP decoration, such catalytic actions are enhanced probably because of the different *d*-band filling in Au@Pd core@shell NP^11,15^.

### Glucose and H_2_O_2_ sensing tests

**Figure 6:**
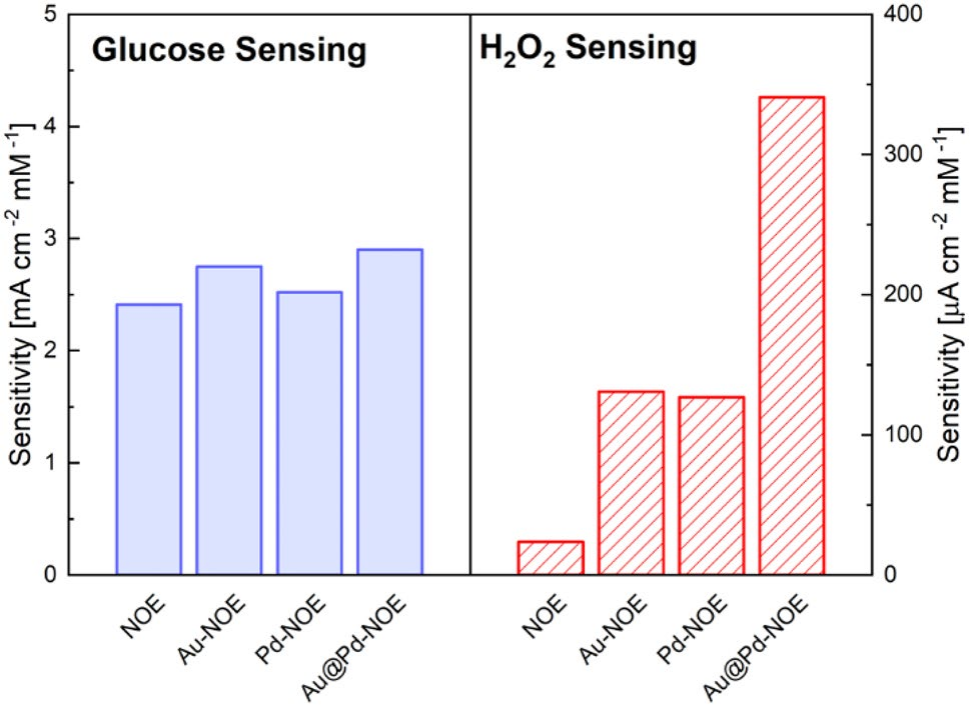
Sensitivity of NOE, Au-NOE, Pd-NOE, and Au@Pd-NOE for glucose and ${\mathrm{H}}_{2}{\mathrm{O}}_{2}$ test.

To validate our model, sensing tests in both oxidant and reducing conditions were conducted, detecting glucose and ${\mathrm{H}}_{2}{\mathrm{O}}_{2}$, respectively. The amperometric responses of NOE, Au-NOE, Pd-NOE, and Au@Pd-NOE samples were recorded by successive additions of glucose or ${\mathrm{H}}_{2}{\mathrm{O}}_{2}$ to electrochemical cell containing 50 mL NaOH (0.1 M). Figure S7 displays typical current–time plot of electrodes with continuous addition of glucose or ${\mathrm{H}}_{2}{\mathrm{O}}_{2}$. Steady-state currents were typically obtained 2–3 s after addition. Concerning glucose test, the fabricated samples show a sensitivity of $2.41, 2.75, 2.52, 2.90\mathrm{ mA c}{\mathrm{m}}^{-2}\mathrm{ m}{\mathrm{M}}^{-1}$ for NOE, Au-NOE, Pd-NOE, and Au@Pd-NOE, respectively. The same electrode, for ${\mathrm{H}}_{2}{\mathrm{O}}_{2}$ test, gave sensitivities of $23.6, 130.9, 127.4, 340\mathrm{ \mu A c}{\mathrm{m}}^{-2}\mathrm{ m}{\mathrm{M}}^{-1}$. Figure 6 summarizes the obtained sensitivities of electrodes, while a large comparison with similar sensors in literature is given in Table S2. No significant changes in sensitivity can be appreciated in glucose test, while for ${\mathrm{H}}_{2}{\mathrm{O}}_{2}$ detection, bimetallic NPs catalyze ${\mathrm{H}}_{2}{\mathrm{O}}_{2}$ reduction with a sensitivity approximately one order of magnitude higher than bare NOE and almost three times higher than samples decorated with monometallic NPs.

These results consolidate our model of energy band bending induced by decoration. Bare NOE (NiO and $\mathrm{Ni}{\left(\mathrm{OH}\right)}_{2})$ is made of *p*-type semiconductor^61,62^, while decorated NOE shows electron reservoirs localized at decoration sites. During glucose oxidation, electrons are transferred from solution to electrode and thus no appreciable effects is observed after decoration. On the other hand, during ${\mathrm{H}}_{2}{\mathrm{O}}_{2}$ reduction, availability of electrons at surface of NOE boosts the sensitivity of decorated electrodes in comparison to bare one. The different orders of magnitude of the sensitivity between the oxidation of glucose and the reduction of ${\mathrm{H}}_{2}{\mathrm{O}}_{2}$ can be easily explained by considering that only in those regions close to NPs (i.e. at the nano Schottky junctions) the electric field induces a modification of energy bands and an accumulation of electrons. As a consequence, only small percentage of surface (with a higher concentration of available electrons) are active in ${\mathrm{H}}_{2}{\mathrm{O}}_{2}$ reduction, while for glucose the whole material is responsible for the oxidation reaction. Moreover, the synergism between Au and Pd, with higher electric field and electron concentration, induces an extra boost in sensitivity and better electrochemical performances than the monometallic counterpart.

## Conclusions

Au, Pd and Au@Pd (core@shell) nanoparticles were synthesized through a low-cost method and used to decorate a Ni oxide nanostructured electrode. The effect of mono- and bimetallic decoration was carefully investigated via electron microscopy and electrochemical analytical techniques, revealing that decoration with core@shell nanoparticles allows a higher catalytic effect both on Ni redox reaction and on H_2_O_2_ sensing tests. The decoration effect was then modeled employing a nano Schottky junction at the nanoparticle–semiconductor interface, leading to a significant energy band bending (extending 5 nm below the decorated side) and to a giant localized electric field (up to ${10}^{8}\mathrm{ V }{\mathrm{m}}^{-1}$), causing a catalysis booster. The bimetallic nanoparticle creates a larger band bending correlated with better catalytic and sensing performances improved by more than a decade in sensitivity. An insight of electron transfer at microscopic scale close to the nanoparticle–semiconductor interface is given.

## Supplementary Information

Supplementary Information 1.

Supplementary Information 2.

## Data Availability

All data generated or analyzed during this study are included in this published article [and its supplementary information files].
