# Supplementary material for: Enlightening the bimetallic effect of Au@Pd nanoparticles on Ni oxide nanostructures with enhanced catalytic activity
Source: Sci Rep. 2023 Feb 24;13:3203. doi: 10.1038/s41598-023-29679-6 (PMC9957992; doi:10.1038/s41598-023-29679-6)
Supplement: Supplementary file 2 — Supplementary Information 2. [file 41598_2023_29679_MOESM2_ESM.docx]

Enlightening the Bimetallic Effect of Au@Pd Nanoparticles on Ni Oxide Nanostructures with Enhanced Catalytic Activity

Luca Bruno^1,2^, Mario Scuderi^3^, Francesco Priolo^1^, Luigi Falciola^4^, Salvo Mirabella^1,2*^

^1^ Dipartimento di Fisica e Astronomia “Ettore Majorana”, Università degli Studi di Catania, via S. Sofia 64, 95123, Catania, Italy.

^2^ IMM-CNR, via S. Sofia 64, 95123, Catania, Italy.

^3^ IMM-CNR, VIII strada 5, 95121, Catania, Italy.

^4^ Dipartimento di Chimica, Università degli Studi di Milano, Via Golgi 19, 20133 Milano, Italy.

^*^Corresponding Author

Email: salvo.mirabella@dfa.unict.it

**Supplementary Information**


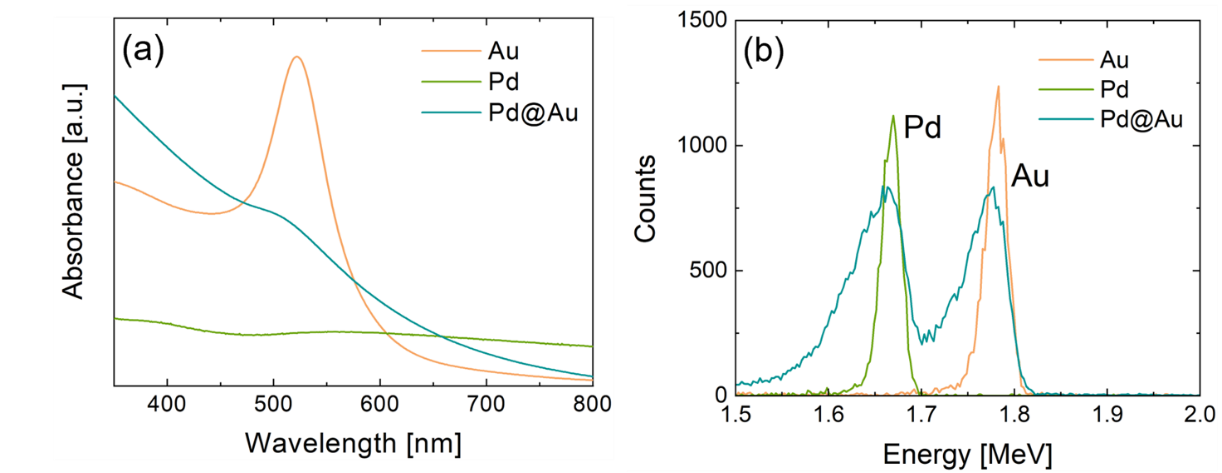


**Figure S1:** (a) Absorbance spectra of Au, Pd and Au@Pd colloidal solutions; (b) RBS spectra of NPs dispersed onto a Si substrate.


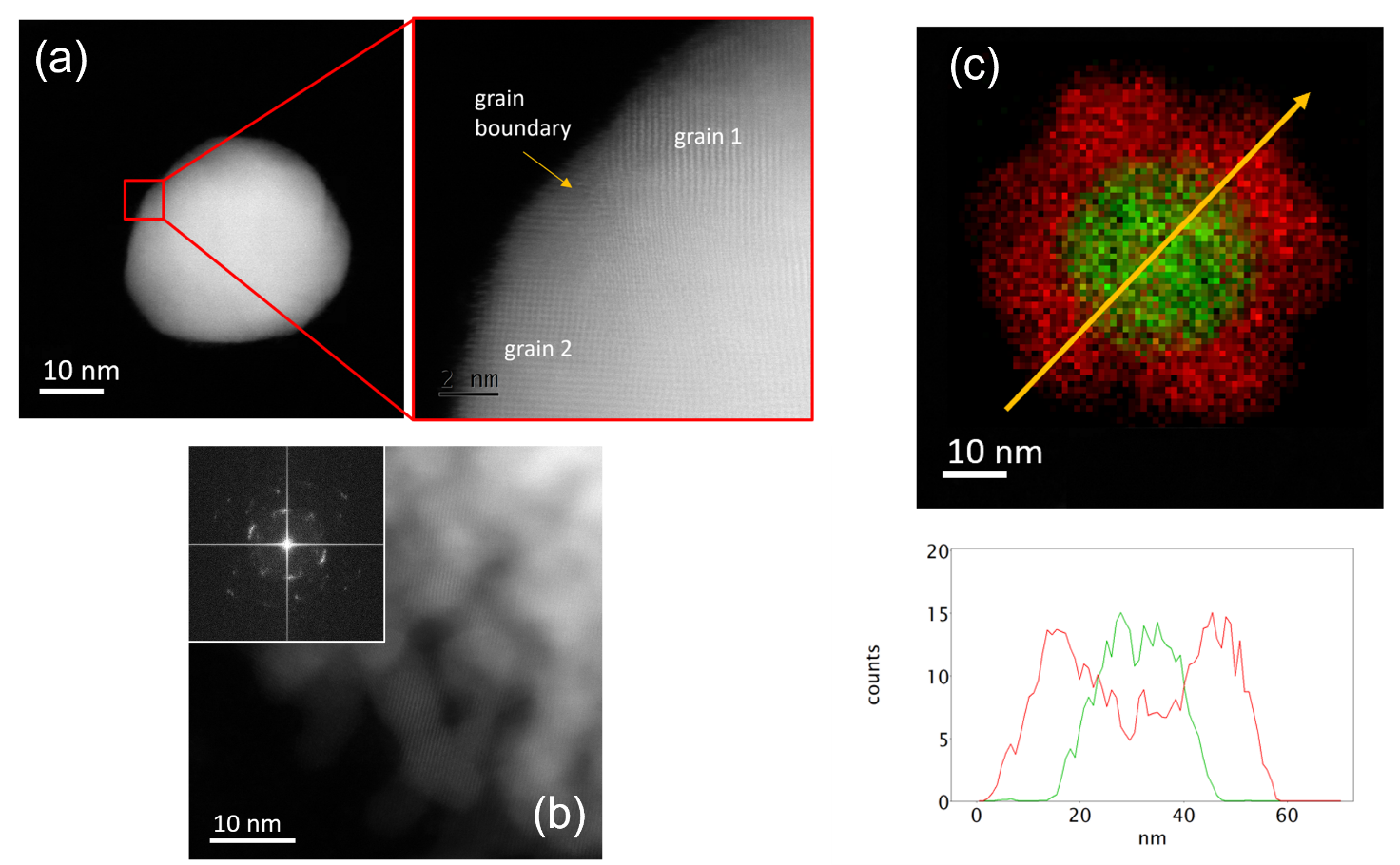


**Figure S2:** (a) STEM micrograph of an Au NP with a corresponding high-magnification micrograph showing a grain boundary; (b) STEM micrograph of the Pd clusters composing the Pt NP and the corresponding FFT showing a polycrystalline pattern; (c) EDX elemental maps of an Au@Pd NP with the corresponding EDX lines scan (Pd red line and Au green line) showing the core shell structure.

**Electrochemical Characterization**

**
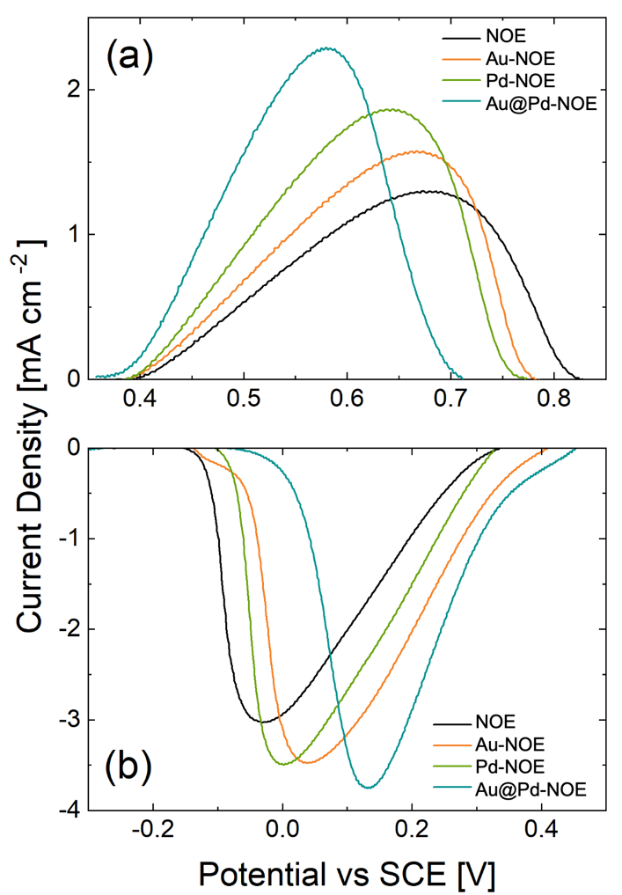
**

**Figure S3:** (a) anodic and (b) cathodic peaks for bare and decorated electrode after the subtraction of the baseline.


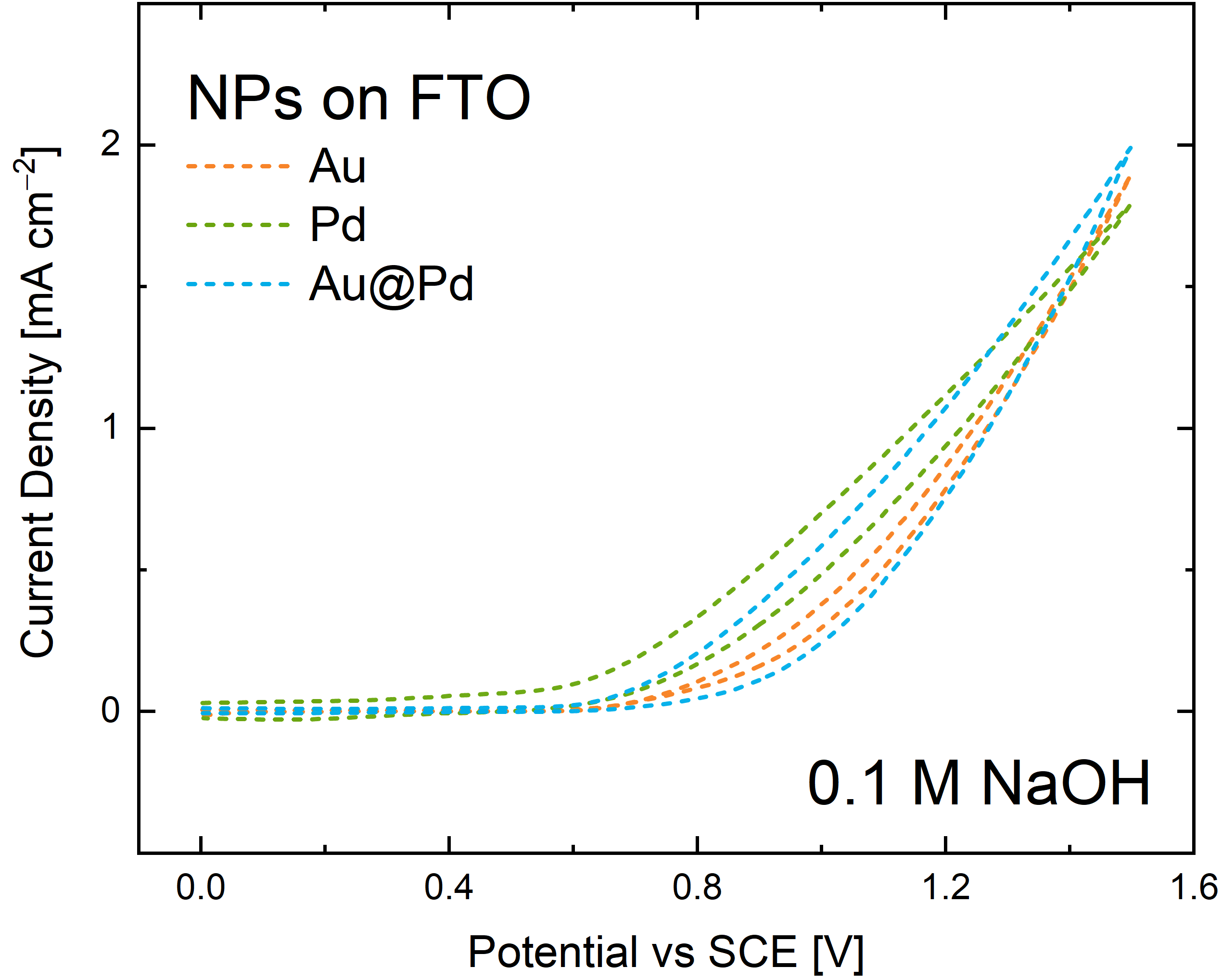


**Figure S4:** Cyclic voltammograms of Au, Pd and Au@Pd NPs on FTO.


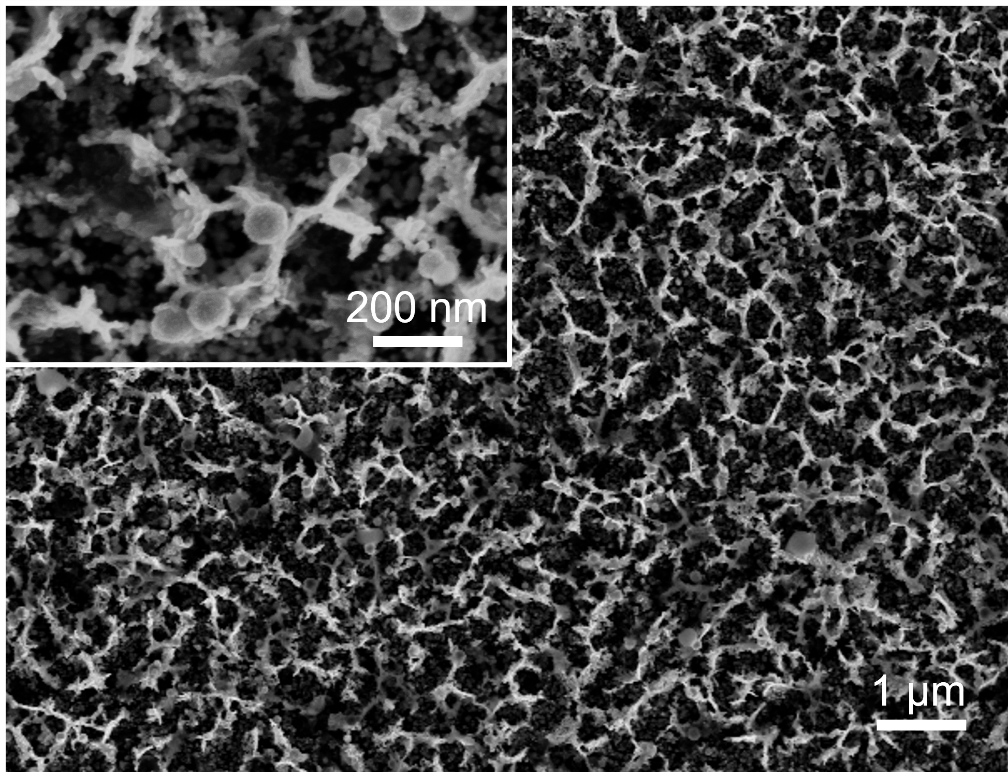


**Figure S5:** SEM image of NOE after electrochemical characterization (Au@Pd NP anchored to NOE in the inset).

Electrochemical Impedance Spectroscopy (EIS) was carried out in the frequency range ${10}^{2}\div{10}^{-1}\mathrm{Hz}$, by applying the peak potential with 5 mV RMS voltage perturbation amplitude, in 0.1 M NaOH solution. The modeling for EIS data with a Randles circuit includes a solution resistance ($R_{S}$) connected in series with a RC circuit, formed by a constant phase element (CPE, to account for the non-ideal capacitive behavior of the surface double layer) in parallel with a charge-transfer resistance ($R_{\mathrm{ct}}$)^1^.

Mott-Schottky analyses were performed by immersing the samples in 0.1 M NaOH, in the potential range $-0.5\div1.0$ V vs SCE with a frequency of 1000 Hz. In a first approximation^2^, using the model of a parallel-plate capacitor, the Mott-Schottky relation can be obtained from^3-6^:

$$\frac{1}{C^{2}}=\frac{2}{\varepsilon_{0}\varepsilon_{r}eN_{D}}\left( E-E_{\mathrm{FB}}-\frac{\mathrm{kT}}{e} \right), (1)$$

in which we assumed that the total capacity is equal to that of the space-charge layer of the material ($C_{\mathrm{SC}}\simeq C$)^4,6^. The obtained M-S plots appear to have a linear section in the potential range around $-0.5\div0.0$ V vs SCE.

| **Sample** | $\mathbf{R}_{\mathbf{S}}\boldsymbol{[\Omega]}$ | $\mathbf{R}_{\mathbf{ct}}\boldsymbol{[\Omega]}$ | $\mathbf{CPE [mF}\mathbf{s}^{\mathbf{n-1}}\mathbf{]}$ | $\mathbf{n}$ |
| --- | --- | --- | --- | --- |
| NOE | $43.7\pm0.3$ | $12.5\pm0.7$ | $1.1\pm0.3$ | $0.53$ |
| Au-NOE | $41.3\pm0.2$ | $10.6\pm0.5$ | $0.7\pm0.2$ | $0.64$ |
| Pd-NOE | $41.2\pm0.3$ | $10.4\pm0.6$ | $0.5\pm0.7$ | $0.65$ |
| Au@Pd-NOE | $40.9\pm0.3$ | $5.6\pm0.6$ | $0.2\pm0.4$ | $0.62$ |

**Table S1:** Circuit parameters calculated from EIS spectra fitting.


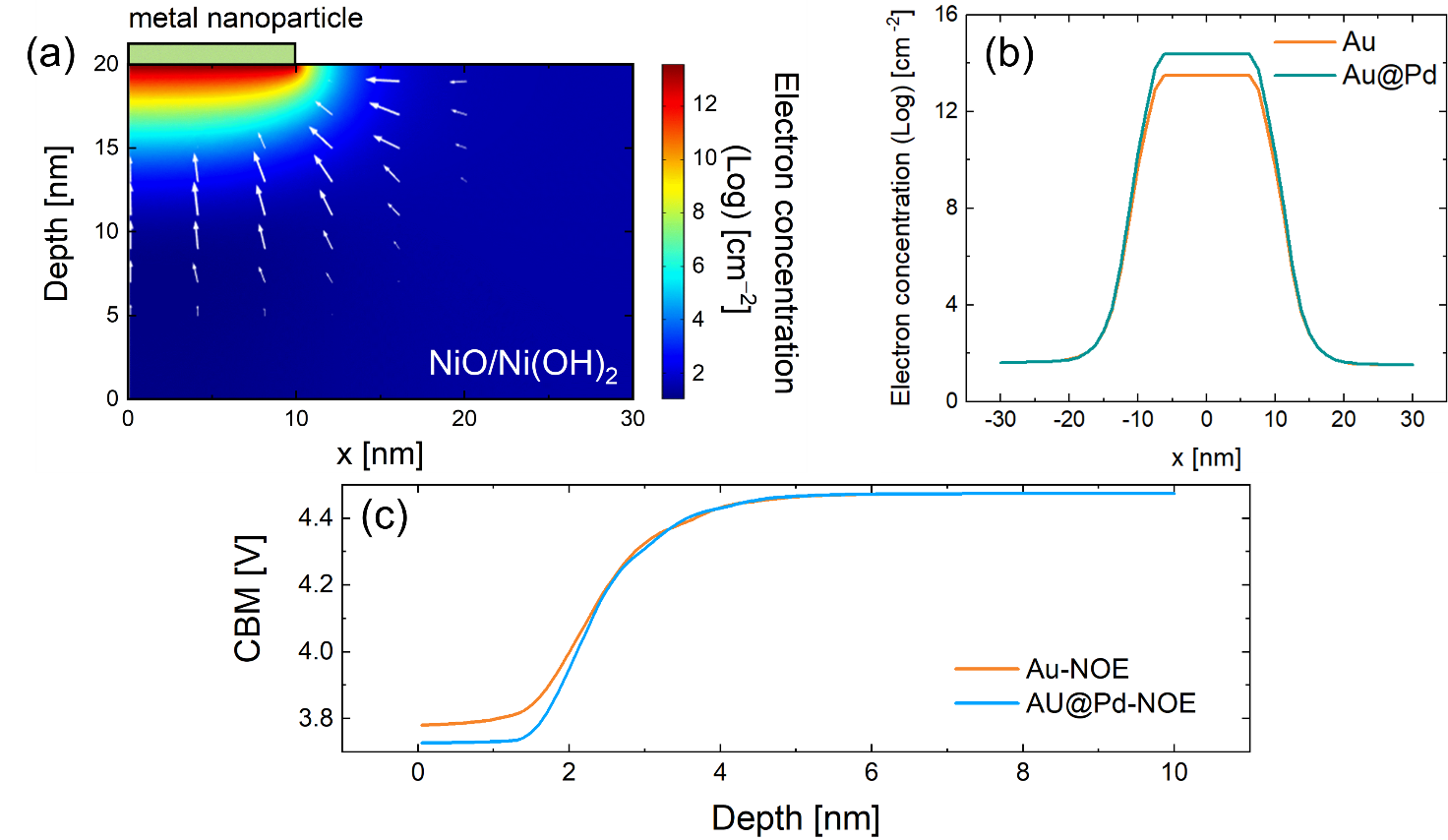


**Figure S6:** (a) 2D COMSOL simulation of the electron concentration at Au@Pd-NOE interface; (b) electron concentration profile at metal-semiconductor interface, and (c) conduction band minimum (CBM) profile for Au and Au@Pd decorated samples.


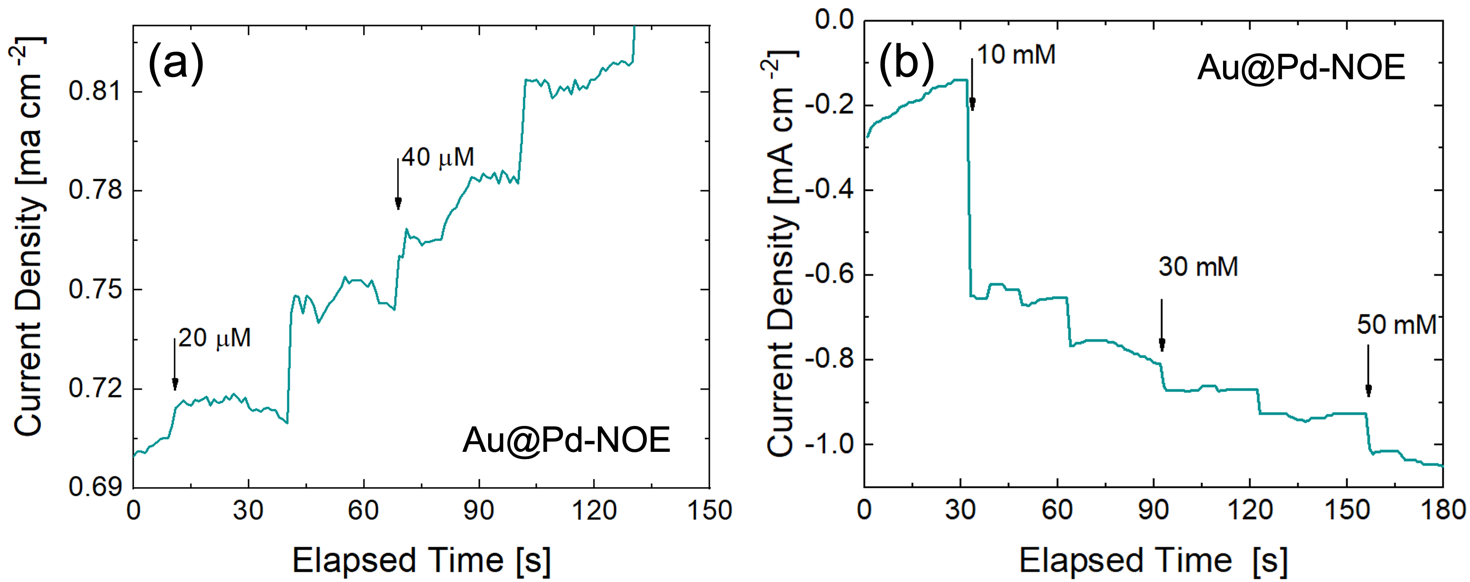


**Figure S7:** Chronoamperometric curves of Au@Pd-NOE sample after subsequent additions of (a) glucose and (b) H_2_O_2_.

| **GLUCOSE** |  |  |  |
| --- | --- | --- | --- |
| **Electrode** | **Sensitivity**  **[**$\boldsymbol{\mu}\mathbf{A c}\mathbf{m}^{\mathbf{-2}}\mathbf{m}\mathbf{M}^{\mathbf{-1}}$**]** | **LOD [m**$\mathbf{M}$**]** | **Ref.** |
| CuO/NiO - Carbon | 856.7 | 0.037 | 7 |
| PtPd - MWCNTs | 112 | 31 | 8 |
| PtPb - MWCNTs | 17.8 | 1.8 | 9 |
| Ag@Cu | 20.9 | 3.5 | 10 |
| Au-CuO - GCE | 3126.76 | 1.4 | 11 |
| Au@Ni/C | 23.17 | 15.7 | 12 |
| Au-Ni | 30.58 | 25 | 13 |
| CuOx/NiOy | 2043 | 0.08 | 14 |
| Au-C | 101.2 | 3 | 15 |
| NiCoP | 6115 | 0.36 | 16 |
| NOE | 2410 | 6 | Our Work |
| Au-NOE | 2750 | 5 | Our Work |
| Pd-NOE | 2523 | 7 | Our Work |
| Au@Pd-NOE | 2901 | 3 | Our Work |
|  |  |  |  |
| **H2O2** |  |  |  |
| **Electrode** | **Sensitivity**  **[**$\boldsymbol{\mu}\boldsymbol{A c}\boldsymbol{m}^{\boldsymbol{-2}}\boldsymbol{m}\boldsymbol{M}^{\boldsymbol{-1}}$**]** | **LOD [m**$\boldsymbol{M}$**]** | **Ref.** |
| PtIr - MWCNTs | 58.8 | 2.5 | 17 |
| PtPd - MWCNTs | 414.8 | 1.2 | 17 |
| PtAu-RGO - GCE | 410.5 | 0.008 | 9 |
| Ag@Cu | 85.1 | 0.3 | 10 |
| Pd@Ag - RGO | 1307.46 | 0.7 | 18 |
| CoFe - NGR | 435.7 | 0.28 | 19 |
| PtCu | 60.4 | 12.1 | 20 |
| PtNi | 208.5 | 31.2 | 20 |
| PtPd | 239.8 | 114 | 20 |
| PtRh | 839.9 | 348 | 20 |
| Au@Pt | 882.2 | 0.1 | 21 |
| Ag-Au | 600 | 0.2 | 22 |
| AuPt | 46.7 | 2.6 | 23 |
| CuOx/NiOy | 271.1 | 0.09 | 14 |
| Au-C | 170.6 | 1.5 | 15 |
| AuPd-NG | 5095.5 | 0.02 | 24 |
| Ag-Au | 260.08 | 0.18 | 25 |
| NOE | 23.6 | 6.7 | Our Work |
| Au-NOE | 130.9 | 1.2 | Our Work |
| Pd-NOE | 127 | 1.3 | Our Work |
| Au@Pd-NOE | 340.8 | 0.5 | Our Work |

**Table S2:** Comparison of analytical performance of bare and decorated Ni NF with recently reported glucose and $H_{2}O_{2}$ sensors.

**References**

1. Randles, J. E. B. Kinetics of rapid electrode reactions. *Faraday Discuss.* (1947) doi:10.1039/DF9470100011.

2. Mora-Seró, I. *et al.* Determination of carrier density of ZnO nanowires by electrochemical techniques. *Appl. Phys. Lett.* (2006) doi:10.1063/1.2390667.

3. Bott, A. W. Electrochemistry of Semiconductors. *Curr. Sep.* (1998).

4. Beranek, R. (Photo)electrochemical methods for the determination of the band edge positions of TiO 2-based nanomaterials. *Advances in Physical Chemistry* (2011) doi:10.1155/2011/786759.

5. Fabregat-Santiago, F., Garcia-Belmonte, G., Bisquert, J., Bogdanoff, P. & Zaban, A. Mott-Schottky Analysis of Nanoporous Semiconductor Electrodes in Dielectric State Deposited on SnO2 Conducting Substrates. *J. Electrochem. Soc.* (2003) doi:10.1149/1.1568741.

6. Hankin, A., Bedoya-Lora, F. E., Alexander, J. C., Regoutz, A. & Kelsall, G. H. Flat band potential determination: Avoiding the pitfalls. *J. Mater. Chem. A* (2019) doi:10.1039/c9ta09569a.

7. Archana, V., Xia, Y., Fang, R. & Gnana Kumar, G. Hierarchical CuO/NiO-Carbon Nanocomposite Derived from Metal Organic Framework on Cello Tape for the Flexible and High Performance Nonenzymatic Electrochemical Glucose Sensors. *ACS Sustain. Chem. Eng.* (2019) doi:10.1021/acssuschemeng.8b05980.

8. Chen, K. J. *et al.* Fabrication and application of amperometric glucose biosensor based on a novel PtPd bimetallic nanoparticle decorated multi-walled carbon nanotube catalyst. *Biosens. Bioelectron.* (2012) doi:10.1016/j.bios.2011.12.020.

9. Cui, H. F. *et al.* Selective and sensitive electrochemical detection of glucose in neutral solution using platinum-lead alloy nanoparticle/carbon nanotube nanocomposites. *Anal. Chim. Acta* (2007) doi:10.1016/j.aca.2007.05.047.

10. Dong, S., Yang, Q., Peng, L., Fang, Y. & Huang, T. Dendritic Ag@Cu bimetallic interface for enhanced electrochemical responses on glucose and hydrogen peroxide. *Sensors Actuators, B Chem.* (2016) doi:10.1016/j.snb.2016.03.129.

11. Felix, S., Grace, A. N. & Jayavel, R. Sensitive electrochemical detection of glucose based on Au-CuO nanocomposites. *J. Phys. Chem. Solids* (2018) doi:10.1016/j.jpcs.2018.06.038.

12. Gao, X. *et al.* Core-shell gold-nickel nanostructures as highly selective and stable nonenzymatic glucose sensor for fermentation process. *Sci. Rep.* (2020) doi:10.1038/s41598-020-58403-x.

13. Lee, W. C. *et al.* Comparison of enzymatic and non-enzymatic glucose sensors based on hierarchical Au-Ni alloy with conductive polymer. *Biosens. Bioelectron.* (2019) doi:10.1016/j.bios.2019.01.028.

14. Long, L., Liu, X., Chen, L., Li, D. & Jia, J. A hollow CuOx/NiOy nanocomposite for amperometric and non-enzymatic sensing of glucose and hydrogen peroxide. *Microchim. Acta* (2019) doi:10.1007/s00604-018-3183-x.

15. Mei, H. *et al.* A nanocomposite consisting of gold nanobipyramids and multiwalled carbon nanotubes for amperometric nonenzymatic sensing of glucose and hydrogen peroxide. *Microchim. Acta* (2019) doi:10.1007/s00604-019-3272-5.

16. Zhu, Y. *et al.* A nickel–cobalt bimetallic phosphide nanocage as an efficient electrocatalyst for nonenzymatic sensing of glucose. *Microchim. Acta* (2020) doi:10.1007/s00604-019-4073-6.

17. Chen, K. J. *et al.* Bimetallic PtM (M=Pd, Ir) nanoparticle decorated multi-walled carbon nanotube enzyme-free, mediator-less amperometric sensor for H 2O 2. *Biosens. Bioelectron.* (2012) doi:10.1016/j.bios.2011.12.037.

18. Guler, M., Turkoglu, V., Bulut, A. & Zahmakiran, M. Electrochemical sensing of hydrogen peroxide using Pd@Ag bimetallic nanoparticles decorated functionalized reduced graphene oxide. *Electrochim. Acta* (2018) doi:10.1016/j.electacta.2018.01.048.

19. Hassan, M., Jiang, Y., Bo, X. & Zhou, M. Sensitive nonenzymatic detection of hydrogen peroxide at nitrogen-doped graphene supported-CoFe nanoparticles. *Talanta* (2018) doi:10.1016/j.talanta.2018.06.003.

20. Janyasupab, M., Liu, C. W., Zhang, Y., Wang, K. W. & Liu, C. C. Bimetallic Pt-M (M = Cu, Ni, Pd, and Rh) nanoporous for H2O 2 based amperometric biosensors. *Sensors Actuators, B Chem.* (2013) doi:10.1016/j.snb.2012.09.099.

21. Li, Z. *et al.* Electrostatic Self-Assembled Bracelet-Like Au@Pt Nanoparticles: An Efficient Electrocatalyst for Highly Sensitive Non-Enzymatic Hydrogen Peroxide Sensing. *ChemElectroChem* (2020) doi:10.1002/celc.202000019.

22. Li, W., Kuai, L., Qin, Q. & Geng, B. Ag-Au bimetallic nanostructures: Co-reduction synthesis and their component-dependent performance for enzyme-free H2O2 sensing. *J. Mater. Chem. A* (2013) doi:10.1039/c3ta00106g.

23. Liu, W. *et al.* Pt and Au bimetallic and monometallic nanostructured amperometric sensors for direct detection of hydrogen peroxide: Influences of bimetallic effect and silica support. *Sensors Actuators, B Chem.* (2018) doi:10.1016/j.snb.2017.08.123.

24. Shang, L., Zeng, B. & Zhao, F. Fabrication of novel nitrogen-doped graphene-hollow AuPd nanoparticle hybrid films for the highly efficient electrocatalytic reduction of H2O2. *ACS Appl. Mater. Interfaces* (2015) doi:10.1021/am507149y.

25. Zhao, L. *et al.* Green Synthesis of Ag–Au Bimetallic Nanoparticles with Alginate for Sensitive Detection of H2O2. *Catal. Letters* (2018) doi:10.1007/s10562-018-2522-1.
